# Engagement Within a Mobile Phone–Based Smoking Cessation Intervention for Adolescents and its Association With Participant Characteristics and Outcomes

**DOI:** 10.2196/jmir.7928

**Published:** 2017-11-01

**Authors:** Raquel Paz Castro, Severin Haug, Andreas Filler, Tobias Kowatsch, Michael P Schaub

**Affiliations:** ^1^ Swiss Research Institute for Public Health and Addiction Zurich University Zurich Switzerland; ^2^ Center for Digital Health Interventions Institute of Technology Management University of St. Gallen St. Gallen Switzerland; ^3^ Energy Efficient Systems Group University of Bamberg Bamberg Germany

**Keywords:** tobacco, alcohol drinking, adolescent, mobile phones, treatment outcome

## Abstract

**Background:**

Although mobile phone–delivered smoking cessation programs are a promising way to promote smoking cessation among adolescents, little is known about how adolescents might actually use them.

**Objective:**

The aim of this study was to determine adolescents’ trajectories of engagement with a mobile phone–delivered smoking cessation program over time and the associations these trajectories have with baseline characteristics and treatment outcomes.

**Methods:**

We performed secondary data analysis on a dataset from a study that compared a mobile phone–delivered integrated smoking cessation and alcohol intervention with a smoking cessation only intervention for adolescents recruited in vocational and upper secondary school classes (N=1418). Throughout the 3-month intervention, participants in both intervention groups received one text message prompt per week that either assessed smoking-related target behaviors or encouraged participation in a quiz or a message contest. Sequence analyses were performed to identify engagement trajectories. Analyses were conducted to identify predictors of engagement trajectory and associations between engagement trajectories and treatment outcomes.

**Results:**

Three engagement trajectories emerged: (1) stable engagement (646/1418, 45.56%), (2) decreasing engagement (501/1418, 35.33%), and (3) stable nonengagement (271/1418, 19.11%). Adolescents who were younger, had no immigrant background, perceived more benefits of quitting smoking, and reported binge drinking preceding the baseline assessment were more likely to exhibit stable engagement. Due to different reach of more engaged and less engaged participants at follow-up, three statistical models (complete-cases, last-observation-carried-forward, and multiple imputation) for the associations of engagement trajectory and smoking outcome were tested. For 7-point smoking abstinence, no association was revealed to be statistically significant over all three models. However, decreasing engagement with the program was associated over all three models, with greater reductions in daily tobacco use than nonengagement.

**Conclusions:**

The majority of tobacco-smoking adolescents engaged extensively with a mobile phone–based smoking cessation program. However, not only stable engagement but also decreasing engagement with a program might be an indicator of behavioral change. Measures to avoid nonengagement among adolescents appear especially necessary for older smokers with an immigrant background who do not drink excessively. In addition, future studies should not only examine the use of specific program components but also users’ engagement trajectories to better understand the mechanisms behind behavioral change.

## Introduction

Tobacco smoking is one of the main contributors to the global burden of disease [1]. A survey of 15- and 16-year-old adolescents covering 36 European countries revealed that 21% considered themselves current smokers [2]. As tobacco use often starts in adolescence, intervening before the development of a substance use disorder gains importance [3].

Mobile phone–based programs for smoking cessation are promising tools for delivering treatment to large numbers of adolescents [3]. Such programs have already been proven more effective than minimal or no intervention in adult smokers [4-8]. Whereas only trends toward the effectiveness of such programs in adolescents have been documented to date [6], studies highlight their acceptance by adolescent smokers with mixed intentions to quit smoking and by adolescent smokers of different genders, educational levels, and immigrant backgrounds [9,10].

Mobile phone–based smoking cessation programs are delivered via apps [11] or texting [10,12], with the greatest difference being the level of engagement demanded by the two approaches. The first demands that users proactively engage with the program, whereas the latter requires users to actively disengage from the program [13]. Engagement, for instance, has been conceptualized in previous studies both as the usage or the subjective experience with the program [14]. For texting-based programs, there is some evidence that the predominant engagers are female [15] and older and that they exhibit lower rates of daily cigarette consumption [16], but none of these studies were conducted on adolescents.

User engagement with different smoking cessation programs has been linked to positive behavioral changes. With Web-based interventions, for instance, higher numbers of visits and page views were associated with abstinence [17-19]. Recent studies on texting-based interventions point in the same direction. In a study by Balmford and Borland [20], the efficacy of a texting-based smoking cessation program was associated with completion of the program. Participants who elected to stop the program were less likely to be abstinent at follow-up. In another study by Heminger et al [15], rather than overall engagement, *postquit* engagement and the use of specific program features such as pledges were specifically predictive of 6-month abstinence. Even more accurately, a study by Christofferson et al [16] identified five different classes of user engagement, which in turn were associated with different levels of interventional success. These investigators extracted two classes of engagement (high engagement and increasing engagement) and three classes of disengagement (rapidly-decreasing engagement, delayed decreasing engagement, and low engagement), demonstrating that participants within the more engaged classes were significantly more likely to be abstinent at weeks 3, 4, and 5 than participants within less engaged classes.

However, there are also studies that question the association between high engagement and positive behavior changes [11,20,21]. For instance, Balmford and Borland [20] found that users with the lowest texting intensity had a greater chance of being abstinent at the 1-month follow-up. The researchers concluded that users tend to be selective as to what they need, which is not to be confused with a lack of motivation. Furthermore, they questioned whether it would be of more help if greater engagement could be achieved among less responsive users. In another pilot study [11] that investigated the use of an app-based smoking cessation program, the total number of actively-viewed quit tips and medication tips was predictive of nonabstinence at 12-week follow-up.

Three methodological issues make the contribution of a user’s level of engagement to long-term abstinence somewhat uncertain. First, only one study has reported long-term outcome associations with engagement [15]. Second, setting a quit date and having a quit attempt is an integral component of most smoking cessation programs [11,15,16,18,20]. Such interventions are typically divided into prequit and postquit phases. There is a lack of studies investigating engagement with a mobile phone–based intervention that was matched to stages of change and did not require subjects to set a quit date. This is of special interest, as most adult and adolescent smokers do not report any serious intention to quit within the next month [10,22]. Third, on the other hand, smokers who enroll in such cessation programs already tend to report an intention to quit smoking [15,16,20], which can lead to a self-selection of more engaged and thus, more successful subjects. To our knowledge, no studies have investigated engagement with a mobile phone–based cessation program in proactively-recruited smokers at different stages of change.

Thus, this study aimed to examine trajectories of program engagement associated with long-term outcomes within a randomized controlled trial (RCT) assessing a fully-automated mobile phone–based program for young smokers that was based on the health action process approach (HAPA) stages of change model [23]. In this study, we conceptualized engagement as the usage of the program. We expected to find trajectories of higher and lower program engagement, similar to the study of Christofferson et al [16]. Compared with their study [16], we did not expect a concrete amount of trajectories, as we applied a different analysis method, and our sample was not only constituted by participants intending to quit smoking. Factors that predict engagement and completion of the 3-month program were analyzed to sort out for whom such programs still need to be improved. We hypothesized that being female [15], older age, and smoking at lower daily rates [16] would predict engagement. In addition, this study investigated adolescents’ engagement with different features of a mobile phone–based intervention, as identifying more and less influential components of such interventions has recently been raised as a means to help refine other health behavior change programs [24].

## Methods

### Participants and Procedures

Data for this study were extracted from a two-arm, parallel-group, cluster RCT that used school class as the randomization unit, as detailed elsewhere [9,25]. Students in vocational or upper secondary schools in Switzerland were invited to participate in a technology-based program called *MobileCoach Tobacco (MCT)* if they (1) either smoked on a daily or occasional basis (at least 4 cigarettes in the preceding month and at least one cigarette during the preceding week) and (2) owned a mobile phone *.* Participating students were reimbursed 10 Swiss francs for participating in the baseline and follow-up assessments and with 0.5 Swiss francs for each of the 11 text message (short service message, SMS) assessments that they answered within the *MCT* program.

In the original trial, the efficacy of an integrated smoking cessation and alcohol intervention (*MCT+*) was tested against a smoking cessation only intervention (*MCT*) for smoking cessation in adolescents. A total of 1471 students from 360 Swiss vocational school classes participated in this study. They were randomly assigned to either the combined program (*MCT+*, n=730) or to the smoking cessation only program (*MCT*, n=741). The original study [9] found no significant difference between the programs in terms of reducing the number of cigarettes used per day (*MCT+* vs *MCT*: −2.7 vs −2.8) or in increasing the 7-day point prevalence of smoking abstinence at follow-up (*MCT+* vs *MCT*: 14.9% vs 14.0%).

The intervention was designed with, and triggered by, the open-source behavioral intervention platform MobileCoach version 1.1 [26]. The original study protocol was approved by the ethics committee of the Faculty of Philosophy at the University of Zurich, Switzerland (date of approval: June 24, 2014). The study was registered at Current Controlled Trials ISRCTN (ISRCTN02427446, assigned September 8, 2014) and executed in full compliance with the Declaration of Helsinki.

### Description of MobileCoach Tobacco

The *MCT+* program combined (1) tailored Web-based feedback on individual drinking behaviors delivered directly after completion of the baseline assessment, (2) tailored mobile phone text messages to promote drinking within low-risk limits over a 3-month period, (3) tailored mobile phone text messages to support smoking cessation for 3 months, and (4) the option of receiving twice daily strategies for smoking cessation centered around a self-defined quit date. Only components (3) and (4) of the integrated intervention were delivered to participants in the *MCT* group. The theoretical backgrounds of these intervention components are described elsewhere [9].

The Web-based feedback, intended only for participants in the combined program, was provided immediately after completion of the baseline assessment. It included individually-tailored information (1) about calorie intake based on personal drinking data and (2) age and gender-specific norms on the number of drinks consumed per week, as well as on the individual’s frequency of binge drinking.

The alcohol-related text messages provided information on (1) strategies for drinking within low-risk limits and (2) the association between smoking and alcohol consumption. These text messages were sent only to those subjects within the *MCT+* condition who reported binge drinking previous to their baseline assessment, where binge-drinking is equivalent to the consumption of 5 or more drinks on a single occasion for men and 4 or more drinks for women. These text messages were sent on Saturdays at 7 PM on even weeks, whereas on odd weeks they were sent at each particular individual’s typical drinking day and time.

The tobacco-related text messages provided information relevant to each subject’s individual HAPA stage of change [23]. On the basis of the HAPA stage [23], subjects can be divided into “preintenders” (individuals with no intention to quit smoking) and “intenders” or “actors” (individuals who seriously intend to quit smoking or have already quit). For preintenders, the text messages addressed the benefits of quitting, risks of smoking, and methods for improving self-efficacy. For intenders, the text messages initiated planning processes, whereas for actors they emphasized self-regulatory skills.

During the 3-month *MobileCoach Tobacco* program, participants in both intervention groups received one text message prompt per week that either assessed smoking-related target behaviors or encouraged the subject’s participation in a quiz or message contest. These prompts were easily answered by typing a single letter, number, or sentence using the mobile phone’s reply function. Every 4 weeks, smoking-related target behaviors, including the person’s HAPA stage of change, were assessed through the question “Have you recently smoked cigarettes?,” with the following response options: (1) “Yes, and I do not intend to quit” (preintender), (2) “Yes, but I am considering quitting” (preintender), (3) “Yes, but I seriously intend to quit” (intender), or (4) “No, I have already quit smoking” (actor). Furthermore, among preintenders, the number of cigarettes smoked per day or week (depending on smoking status: daily or occasionally) was assessed every 4 weeks. For intenders and actors, the use of strategies to cope with craving, which were individually chosen within the baseline assessment, was assessed: for example, “Did you apply the following strategy recently? When I am at a party, I distract myself from smoking by dancing. Yes (Y) No (N).”

Quizzes were included thrice during the *MCT,* with the questions targeting (1) smoking norms (percentage of smokers within the same age- and gender-specific reference group), (2) the health consequences of smoking cessation (days until positive health consequences after smoking cessation), and (3) expenditures on cigarettes (money spent on cigarettes per year).

A contest that required participants to create a text message to motivate other participants to quit smoking (for nonintenders) or to provide concrete ways to help others quit smoking (for intenders and actors) was conducted twice during the intervention period. The best text message from each of the two categories, rated weekly by a tobacco cessation expert, was sent anonymously to participants in the respective categories after 48 hours.

Finally, additional text messages were offered to subjects who reported having the intention to quit smoking. Intenders and actors were informed biweekly about the option of receiving additional information around a chosen quit date. After entering a scheduled quit date, the program provided up to 2 daily text messages on quit-day preparation and relapse prevention (weeks −1 to +1: 2 daily text messages; weeks +2 and +3: one daily text message).

### Measures

Participants took part in a Web-based health survey during a regular class session, by which data on potential predictors of engagement and outcome variables were collected. The sociodemographic characteristics that were assessed were gender, age, educational attainment, and immigrant background. The following common Swiss levels of educational attainment were assessed: (1) none, (2) secondary school, (3) extended secondary school, and (4) technical or high school. We assessed the country of birth of both parents of the students to identify any potential immigrant background. On the basis of this information, participants were assigned to one of the following categories: (1) neither parent born outside Switzerland, (2) one parent born outside Switzerland, or (3) both parents born outside Switzerland. For further analysis, we grouped subjects with either a one- or two-sided immigrant background into a single category for comparison against nonimmigrants.

The health-related variables that were assessed were perceived stress, physical activity, body weight, typical number of drinks consumed per week, and whether any binge drinking had occurred in the month before the baseline assessment. Perceived stress was measured using the following single item: “In the last month, how severely have you felt stressed?” Participants were asked to indicate their response on a 6-point Likert scale that ranged from *not at all* to *very*. Self-reported moderate to vigorous physical activity was measured by a question derived from the Health Behaviour in School Aged Children study [27]: “Outside school, how many hours a week do you exercise or participate in sports that make you sweat or out of breath?” The typical number of drinks consumed weekly was assessed via a 7-day drinking calendar similar to the Daily Drinking Questionnaire [28], for which participants were asked to think about a typical week in the preceding month and record the number of standard drinks they typically consumed each day during that week. Examples of standard drinks containing 12 g to 14 g of ethanol were provided for beer, wine, spirits, alcopops, and cocktails, along with conversion values (eg, three 0.5 L cans of beer=6 standard drinks). Binge drinking was assessed by asking participants to report the number of standard drinks they consumed on their heaviest drinking occasion over the preceding 30 days.

Tobacco smoking status was assessed using the question, “Are you currently smoking cigarettes?” with the following response options: (1) “Yes—I smoke cigarettes daily,” (2) “Yes—I smoke cigarettes occasionally, but not daily,” and (3) “No.” In occasional smokers, we assessed the number of days they typically smoked per month, the total number of cigarettes smoked within the previous 7 days, and the number of cigarettes smoked on a typical smoking day. In daily smokers, we only assessed the mean number of cigarettes smoked per day. For occasional smokers, the average number of cigarettes smoked per day was computed by multiplying the typical number of smoking days per month with the number of cigarettes smoked on a typical smoking day and dividing this product by 30.

Additionally, we assessed the following smoking-related variables: HAPA stage of change and the number of previous quit attempts. Each subject’s HAPA stage of change was assessed using the following question: “Have you recently smoked cigarettes?” with the following response options (1) “Yes, and I do not intend to quit” (preintenders), (2) “Yes, but I am considering quitting” (preintenders), and (3) “Yes, but I seriously intend to quit” (intenders). Subjects were asked about their previous attempts to quit smoking with the question—“Have you ever made a serious attempt to quit smoking?”—for which they were provided the response options “No,” “Yes—once,” and “Yes—more than once.”

Engagement with the program was assessed in terms of the number of program participants who unsubscribed from the program (program attrition), the number of responses to the weekly text message prompts, the percentage of retrieved versus sent media objects within the program, and the number of smokers who entered a quit date and activated the additional quit day preparation program.

Smoking behavior at 6-month follow-up was assessed as the (1) 7-day point prevalence of smoking abstinence and (2) the mean number of cigarettes smoked per day. To assess the 7-day point prevalence of smoking abstinence, subjects were asked to indicate whether they had taken a puff of a cigarette within the 7 days previous to follow-up. The mean number of cigarettes smoked per day was assessed and computed in the same way as at the baseline assessment.

### Statistical Analyses

As a first step, we analyzed whether persons who actively unsubscribed from the intervention differed from those who remained in the intervention, applying Pearson chi-square analysis to examine differences in categorical variables and unpaired student *t* tests for continuous variables. Given that the combined intervention was more extensive, we also examined whether program attrition differed as a function of study condition. Participants who had either unsubscribed or did not receive the text messages, as seen from program log files, were excluded from further analysis. We then explored the use of different program features for the total sample and by treatment arm.

Subsequently, we examined engagement trajectories by analyzing answers to weekly prompts, which were identical for both study groups. To this end, we performed sequence analysis using the TraMineR library (version 1.8-8) in R [29]. For each participant, answers to prompts (as described previously) were ordered into a sequence of states (ie, engagement trajectories). Similarities between participants’ state sequences were computed using the optimal matching (OM) distance algorithm. OM is defined as the minimal effort, in terms of insertions, deletions, and substitutions, of transforming one sequence into another. Homogeneous engagement trajectory groups (clusters) were then constructed from the distance matrix using agglomerative nesting hierarchical clustering and Ward’s linkage method. The number of clusters chosen was based on the highest relative loss of inertia (see function HCPC in FactoMineR package [30]) and upon the quality of the clusters according to the average silhouette width (ASW) [31]. The ASW ranges from −1 to +1 and can be interpreted as the degree of coherence among assignments to clusters: a high degree of coherence (close to 1) indicates large between-group distances and strong within-group homogeneity.

Upon detecting different engagement trajectories, we examined for baseline differences between the clusters. Subsequently, we conducted multinomial logistic regression analysis to identify predictors of clusters characterized by lower engagement trajectories, compared with those with higher engagement trajectories. Initially, separate univariate multinomial logistic regression analyses were performed (subsequently referred to as univariate analyses) to evaluate potential predictors of engagement trajectories. After these univariate analyses, multivariate prediction models were developed. As suggested by Hosmer et al [32], variable selection consisted of the following steps: (1) significant predictors (*P*<.05) identified during univariate analyses were entered into the preliminary multivariate model; (2) variables that were nonsignificant at *P*>.05 were removed, one at a time, starting with those with the highest *P* values (backward selection); and (3) to account for suppressor effects, the resulting model was verified by adding the aforementioned excluded variables, separately, to the regression model. Only variables that were significant at *P*<.05 were retained in the final multinomial regression model (forward selection).

Finally, we compared smoking outcomes between participants grouped by their engagement trajectory. As participants were nested in school classes, we conducted a generalized linear mixed model for the 7-day point prevalence of abstinence. For changes in consumed cigarettes per day, we conducted a linear mixed model. Engagement trajectory was included as an independent variable (fixed effect) and school class as a single random effect (random intercept). These analyses were conducted using the lme4 library (version 3.2.1) in R [33] on the following three statistical models because of the disparate reach of more engaged than less engaged participants at follow-up: (1) a complete case analysis (CCA) dataset, (2) a last-observation-carried-forward (LOCF) dataset, and (3) an intention-to-treat (ITT) dataset. Details of outcome analysis and missing data imputation procedures are provided in Haug et al [9]. R version 3.3.3 (The R Foundation for Statistical Computing) was used to perform all sequence analyses and outcome analyses, whereas the Statistical Package for the Social Sciences (SPSS) version 22 (IBM Corp) was used for all other analyses. All statistical tests were two-tailed, with *P*<.05 set as the criterion for statistical significance.

## Results

### Participants

**Figure 1:**
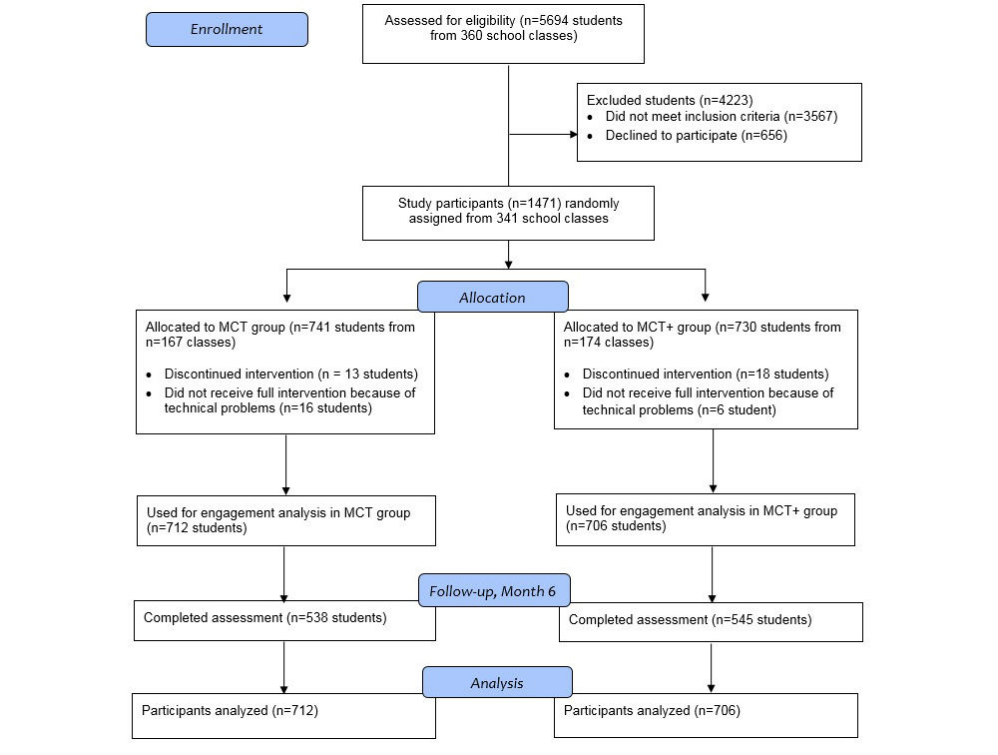
Participants’ progress through the trial.

Figure 1 depicts the progression of participants through the trial. Of the original 1471 study participants, 1418 (96.39%) completed the program. Those who failed to complete their intervention had either signed off (31/1471, 2.11%) or discontinued the intervention because of technical problems (22/1471, 1.49%). No significant baseline differences were observed between those who did and did not complete the intervention. Program attrition also did not differ between the two treatment arms, with 13 of the 741 (1.8%) participants in the *MCT* choosing to unsubscribe compared with 18 of the 730 (2.5%) assigned to the *MCT+* (χ^2^_2_=0.9, *P=*.34). Of the 1418 participants analyzed for this study, 863 (60.86%) were female. The reported mean age was 18.6 (standard deviation [SD] 3.1). More than half (740/1418, 52.18%) reported either a one-sided or two-sided immigrant background, and almost all (1180/1418, 83.21%) had reached at least the lowest educational degree (ie, secondary school). Two-thirds of the sample (1083/1418, 76.37%) took part on the follow-up assessment; 538 of the 712 (75.6%) participants were assigned to the *MCT* and 545 of the 706 (77.2%) were assigned to the *MCT+*.

### Use of Different Program Features

Table 1 summarizes different program use characteristics across the total sample and by intervention group. Participants answered a mean of 6.6 (SD 3.5) out of 11 text message prompts. Each participant received between 3 and 5 text messages containing media objects (videos and pictures) that had to be downloaded. On average, participants downloaded 20.5% (SD 31.5) of the received media content. Participants in the *MCT+* downloaded media content significantly more often than their *MCT* counterparts (23.6% vs 17.9%, *P*<.001). Roughly half of the subjects answered all or almost all of their text message prompts. The fewest answers were recorded for the contest prompt at week 8 (24.9%) and for the HAPA stage query at week 10 (42.9%).

### Engagement Analysis

**Figure 2:**
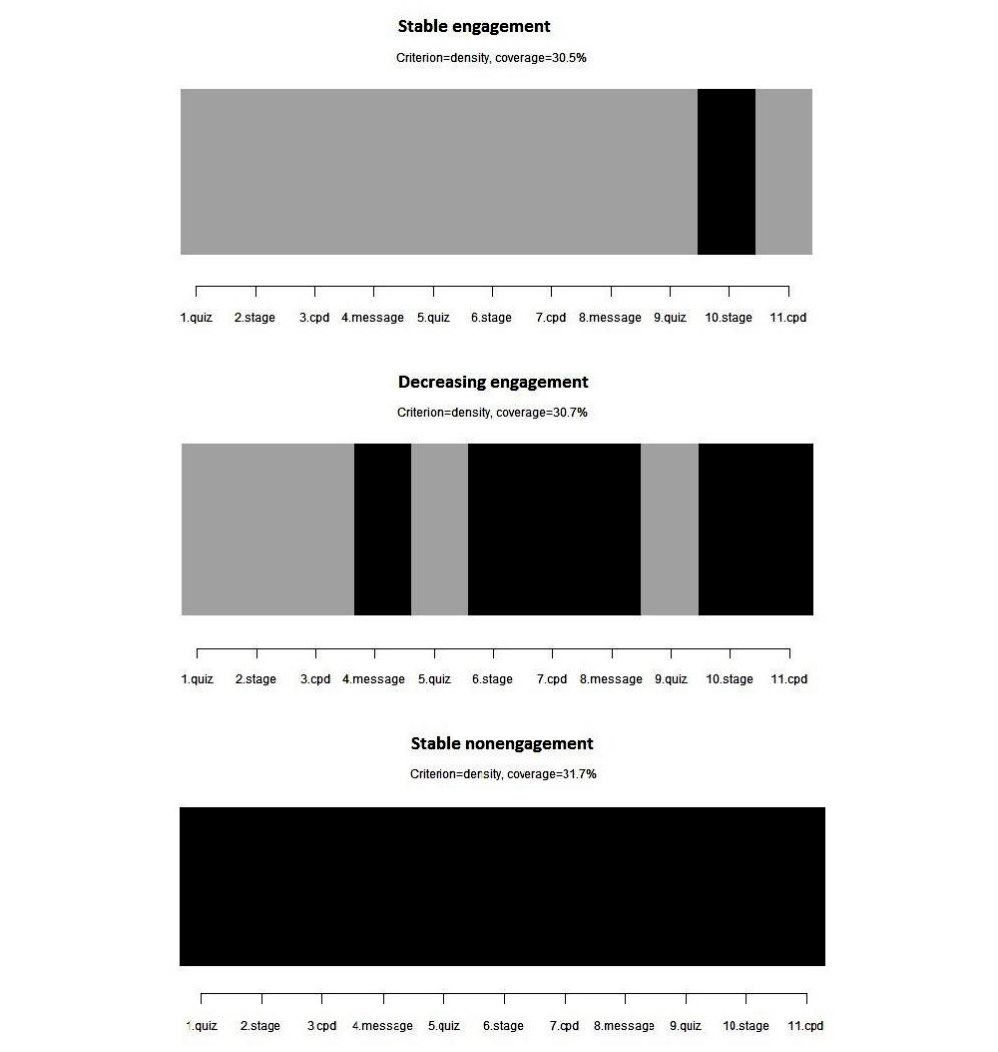
The prototype engagement trajectory within each cluster. Columns represent the 11 prompts that could be answered by the participants. Black boxes represent nonreplies, and gray boxes represent replies.

Our inspection of answer behavior over the 3-month intervention revealed different types of engagement trajectory. Some participants exhibited a stable answer pattern (either usually answered or almost never answered text messages). Other participants displayed irregular trajectories. The highest relative loss of inertia measure suggested the following three-cluster solution: cluster 1=stable engagement (SE), cluster 2=decreasing engagement (DE), and cluster 3=stable nonengagement (SNE). On the basis of the ASW, the quality of the three clusters ranged from poor (cluster 2=−0.02) to good (cluster 1=0.55) and excellent (cluster 3=0.70). The low ASW for cluster 2 was because the engagement trajectories included within the cluster differed to a great extent. Some subjects answered text messages only in the beginning, whereas others answered depending on the topic. There were also some participants who only started to answer text messages at the end of the program (Figure 2). As the common element within all these trajectories included in cluster 2 is their instability, the three-cluster solution was considered adequate for the purposes of this study.

**Table 1:** Use of program components by the overall study sample and by study group.

Program components		All (N=1418)	*MCT* (n=712)	*MCT+* (n=706)	*P* value
Questions answered, mean (SD^a^)^b,c^		6.6 (3.5)	6.5 (3.6)	6.8 (3.5)	.22
Percentage of media objects viewed or of media objects sent, mean (SD)^b,c^		20.8 (31.5)	17.9 (32.6)	23.6 (30.0)	<.001
**Answer to quizzes^d,e^, n (%)**					
	Quiz costs (week 1)	975 (68.76)	481 (67.6)	494 (70.0)	.33
	Quiz health (week 5)	898 (63.32)	438 (61.5)	460 (65.2)	.16
	Quiz norms (week 9)	863 (60.86)	429 (60.3)	434 (61.5)	.64
**Answer to HAPA^f^stage of change^d,e^, n (%)**					
	Stage 1 (week 2)	1206 (85.04)	598 (83.9)	608 (86.1)	.26
	Stage 2 (week 6)	975 (68.76)	485 (68.7)	490 (68.8)	.96
	Stage 3 (week 10)	609 (42.94)	295 (41.4)	314 (44.5)	.25
**Answer to smoking-related questions^d,e^, n (%)**					
	CPD^g^ or CPW^h^ or coping strategy (week 3)	992 (69.95)	490 (68.8)	502 (71.1)	.35
	CPD or CPW or coping strategy (week 7)	876 (61.77)	444 (62.4)	432 (61.2)	.65
	CPD or CPW or coping strategy (week 11)	749 (52.82)	361 (50.7)	388 (55.0)	.11
**Answer to contest, n (%)^d,e^**					
	Motivational or quit contest (week 4)	626 (44.14)	303 (42.6)	323 (45.8)	.23
	Motivational or quit contest (week 8)	353 (24.89)	175 (24.6)	178 (25.2)	.78
Setting of a quit date^d,e^		156/475 (32.8)	79/239 (33.1)	77/236 (32.6)	.92

^a^SD: standard deviation.

^b^*t* test.

^c^Degrees of freedom=1416.

^d^Chi-square test.

^e^Degrees of freedom=1.

^f^HAPA: health action process approach.

^g^CPD: cigarettes smoked per day.

^h^CPW: cigarettes smoked per week.

**Figure 3:**
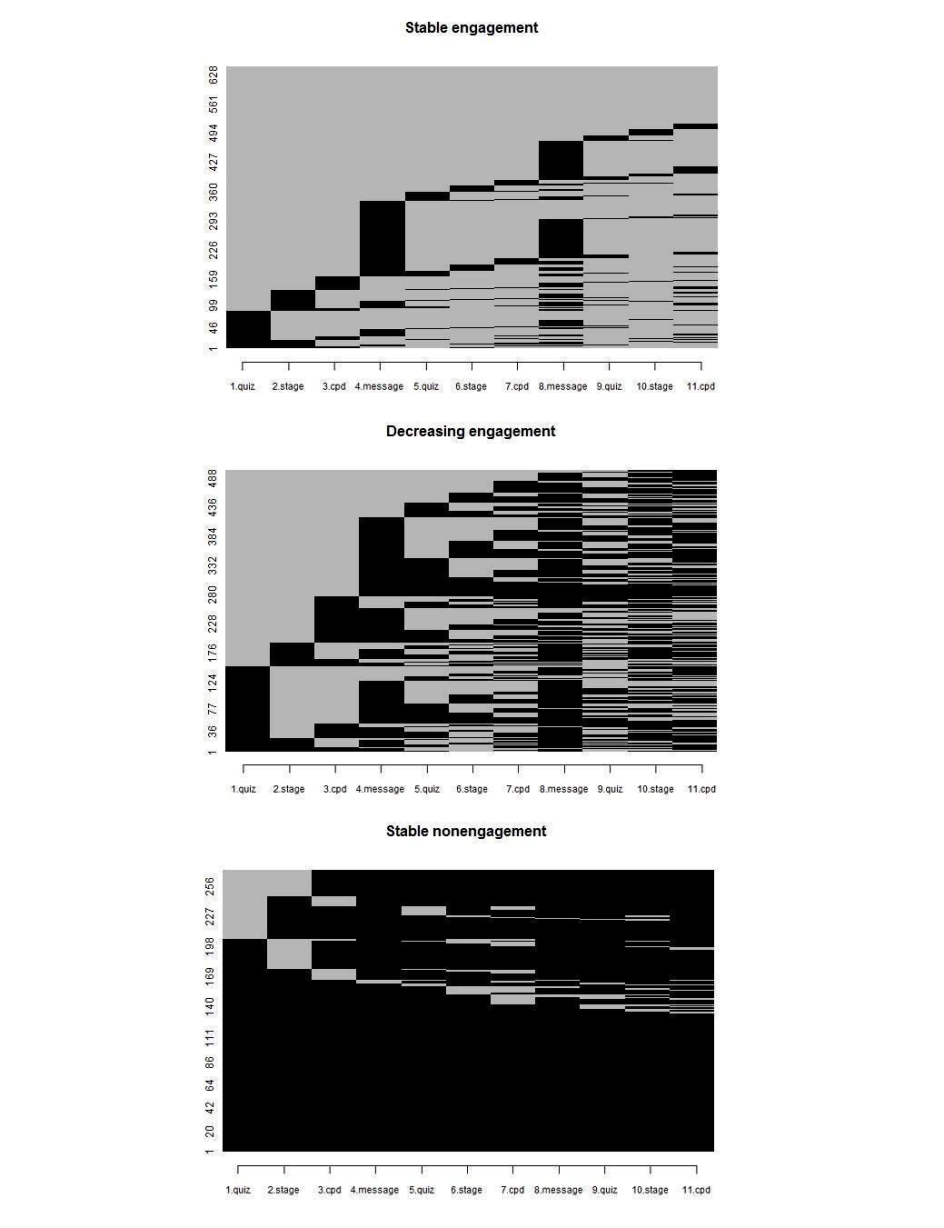
Individual engagement trajectories within each of the three clusters. Rows represent participants and columns represent the 11 prompts that could be answered by the participants. Black boxes represent nonreplies, and gray boxes represent replies.

Figures 2 and 3 describe the three clusters in different ways. The first figure displays the response or nonresponse of individuals to each of the 11 prompts within the different clusters. Figure 2 highlights the prototype engagement trajectory within each of the three clusters. The typical participant within cluster 1 (SE) answered to almost all text messages, except for the second request to send their own message to motivate other participants to quit smoking or remain cigarette free. The typical participant within cluster 3 (SNE) did not respond to any of the prompts. Meanwhile, the typical participant within cluster 2 (DE) did not reply to the two message contests and exhibited a steadily decreasing response rate. This last pattern is associated with the repetition of questions, such as queries relating to the person’s HAPA stage of change and cigarettes per day or per week.

### Predictors of Engagement Trajectory

Table 2 summarizes the baseline characteristics of participants by engagement trajectory. There were significant differences between the three clusters with regard to age (*P*=.006), immigrant background (*P*<.001), educational attainment (*P*=.04), binge drinking (*P*<.001), HAPA stage of change (*P*<.05), and self-perceived benefits of quitting (*P*<.001).

Table 3 shows which of the aforementioned variables were predictive of engagement trajectory within the multivariate model. Being older (Odds ratio [OR]=1.05, *P*=.04) and having an immigrant background (OR=0.76, *P*=.02) predicted a decreasing engagement with the program compared with a stable engagement. Furthermore, participants who perceived more benefits of quitting were more likely to display stable than decreasing engagement with the program (OR=0.52, *P*=.007). Compared with stable engagement, nonengagement was predicted by immigrant background (OR=0.47, *P*<.001) and binge-drinking behavior (OR=1.54, *P*=.005). Being a stable nonengager was more likely than being a stable engager, when participants reported an immigrant background and no binge drinking within the month previous to baseline.

### Engagement Trajectories and Smoking Behavior

Treatment outcome by type of engagement trajectory and comparisons of outcomes between engagement trajectories are summarized in Table 4. Reach at follow-up differed importantly between stable engagers (84.3%, 545/646), decreasing engagers (74.5%, 373/501), and nonengagers (59.0%, 160/271). Due to this, three statistical models were tested. Only the reduction in cigarettes per day among decreasing engagers differed significantly from stable nonengagers under the CCA, LOCF, and ITT assumptions (CCA: beta=.65, *P*=.02; LOCF: beta=.43, *P*=.01; and ITT: beta=.54, *P*=.03). Decreasing engagers smoked significantly fewer cigarettes per day at the end of the intervention than nonengagers.

With respect to the 7-day point prevalence of abstinence at 6-month follow-up, no comparison revealed a significant difference under all three assumptions. On ITT analysis, the odds of being abstinent at follow-up was higher among nonengagers than engagers (OR=1.32, *P=*.02). But this finding must be interpreted with caution, as bias in the multiple imputation of missing data seems probable because of the different amount of available information at follow-up. Caution is also warranted, as under the missing-as-smoker assumption, the odds for being abstinent turn in the opposite direction.

**Table 2:** Demographic and health behavior characteristics of the study sample by engagement trajectory.

Demographic characteristics		All (N=1418)	Stable engagement (n=646)	Decreasing engagement (n=501)	Stable nonengagement (n=271)	*P* value
**Intervention group^a,c^, n (%)**						
	MCT^g^	712 (50.21)	319 (49.4)	248 (49.5)	145 (53.5)	.48
	MCT+^h^	706 (49.78)	327 (50.6)	253 (50.5)	126 (46.5)	
Female sex^a,c^, n (%)		863 (60.86)	398 (61.6)	293 (58.5)	172 (63.5)	.35
Immigrant background^a,c^, n (%)		740 (52.18)	293 (45.4)	268 (53.5)	179 (66.1)	<.001
Age in years, mean (standard deviation [SD^f^])^b,c^		18.6 (3.1)	18.4 (2.8)	18.8 (3.4)	18.9 (2.9)	.006
**Educational level^a,e^, n (%)**						
	Secondary school	1180 (83.21)	555 (85.9)	401 (80.0)	224 (82.7)	.04
	Vocational school	189 (13.32)	67 (10.4)	86 (17.2)	36 (13.3)	
	Technical or high school or university	30 (2.11)	13 (2.0)	10 (2.0)	7 (2.6)	
	Unknown	19 (1.33)	11 (1.7)	4 (0.8)	4 (1.5)	
Hours of moderate to vigorous extracurricular physical activity per week, mean (SD)^b,c^		3.5 (3.6)	3.4 (3.4)	3.7 (3.8)	3.2 (3.6)	.08
Number of alcoholic drinks consumed per week, mean (SD)^b,c^		9.9 (12.1)	10.2 (12.1)	10.3 (11.8)	8.4 (12.5)	.09
**Binge drinking^a,c^, n (%)**						
	No	465 (32.79)	191 (29.6)	158 (31.6)	116 (42.8)	<.001
	Yes	952 (67.13)	455 (70.4)	342 (68.4)	155 (57.2)	
**Tobacco smoking status^b^, n (%)**						
	Daily smoker	1075 (75.81)	476 (73.7)	390 (77.8)	209 (77.1)	.22
	Occasional smoker	343 (24.18)	170 (26.3)	111 (22.2)	62 (22.9)	
Number of cigarettes smoked per day, mean (SD)^b,c^		10.1 (7.3)	9.9 (7.3)	10.5 (7.4)	10.0 (7.1)	.32
**Stage of change^a,d^, n (%)**						
	No intention to quit	396 (27.92)	200 (31.0)	124 (24.8)	72 (26.8)	.03
	Considering quitting	825 (58.18)	372 (57.6)	291 (58.2%)	162 (60.2)	
	Serious intention to quit	194 (13.68)	74 (11.5)	85 (17.0)	35 (13.0)	
Benefits of quitting smoking, mean (SD)^b,c^		1.38 (0.3)	1.41 (0.3)	1.36 (0.3)	1.37 (0.3)	<.001
**Previous quit attempts^a,d^, n (%)**						
	None	507 (35.75)	247 (38.2)	173 (34.6)	87 (32.5)	.10
	One	608 (42.87)	276 (42.7)	205 (41.0)	127 (47.4)	
	Two or more	299 (21.08)	123 (19.0)	122 (24.4)	54 (20.1)	

^a^χ^2^ test.

^b^*F* value.

^c^Degrees of freedom=2.

^d^Degrees of freedom=4.

^e^Degrees of freedom=6.

^f^SD: standard deviation.

^g^MCT: smoking cessation only program.

^h^MCT+: integrated smoking cessation and alcohol intervention.

**Table 3:** Predictors of engagement trajectory. *R*^2^=.04 (Cox and Snell) and *R*^2^=.05 (Nagelkerke). Model χ^2^_8_=59.8, *P*<.001.

Predictors of engagement trajectory		Beta (SE^a^)	*P* value	OR^b^ (95% CI)
**Stable engagement (ref^c^) versus decreasing engagement**				
	Intercept	−.05 (.57)	.93	
	Age in years	.05 (.02)	.04	1.05 (1.003-1.09)
	Immigration background (ref yes)	−.28 (.12)	.02	0.76 (0.59-0.96)
	Binge drinking (ref yes)	.01 (.13)	.98	1.00 (0.78-1.30)
	Benefits of quitting smoking	−.66 (.24)	.007	0.52 (0.32-0.84)
**Stable engagement (ref) versus stable nonengagement**				
	Intercept	−1.07 (.68)	.12	
	Age in years	.05 (.03)	.05	1.05 (0.99-1.10)
	Immigration background (ref yes)	−.76 (.15)	<.001	0.47 (0.35-0.63)
	Binge drinking (ref yes)	.43 (.15)	.005	1.54 (1.14-2.08)
	Benefits of quitting smoking	−.37 (.29)	.21	0.70 (0.39-1.24)

^a^SE: standard error.

^b^OR: odds ratio.

^c^ref=reference category.

**Table 4:** Comparison of treatment outcomes between different engagement trajectories. Descriptive data are based on complete cases. Test value for continuous outcome= *t*-value; for dichotomous outcome=z value.

Engagement trajectory	Mean (SD)	Difference in cigarettes per day^a^			n (%)	7-day point prevalence of smoking abstinence^b^		
		CCA^c^ (*P* value)	LOCF^d^ (*P* value)	ITT^e^ (*P* value)		CCA (*P* value)	MAS^f^ (*P* value)	ITT (*P* value)
								
SE^g^ (ref^h^)	2.36 (5.5)	.25 (.34)	−.19 (.24)	.07 (.77)	73 (13.4)	1.18 (.16)	0.96 (.76)	1.32 (.02)
SNE^i^	2.43 (5.6)				29 (18.1)			
SE (ref)	2.36 (5.5)	.52 (.01)	.14 (.32)	.40 (.04)	73 (13.4)	1.05 (.58)	0.97 (.77)	1.11 (.21)
DE^j^	3.44 (7.1)				54 (14.5)			
SNE (ref)	2.43 (5.6)	.65 (.02)	.43 (.01)	.54 (.03)	29 (18.1)	0.91 (.46)	0.99 (.97)	0.87 (.25)
DE	3.44 (7.1)				54 (14.5)			
SE (ref)	2.36 (5.5)	.25 (.046)	.00 (.99)	.17 (.18)	73 (13.4)	1.06 (.31)	0.98 (.74)	1.09 (.08)
SNE and DE	2.94 (6.4)				83 (15.6)			

^a^beta

^b^odd ratio.

^c^CCA: complete-case dataset.

^d^LOCF: last-information-carried-forward.

^e^ITT: intention-to-treat dataset.

^f^MAS: missing-as-smoker.

^g^SE: stable engagement.

^h^ref.: reference category.

^i^SNE: stable nonengagement.

^j^DE: decreasing engagement.

## Discussion

### Principal Findings

Using a proactively-recruited sample of smoking adolescents with mixed intentions to quit smoking, this study examined (1) the use of different components of a mobile phone–based smoking cessation program, (2) different prototypes of engagement trajectory, (3) the association between engagement trajectories and adolescent characteristics, and (4) the association between engagement trajectories and treatment outcomes.

The main findings are as follows: (1) the components of the mobile phone–based smoking cessation program were used over the 3-month intervention in a regular way, with quizzes being the component with the highest participation rate and repeated smoking-related assessments the least-used component, (2) three distinct engagement trajectories emerged: two characterized by higher levels of engagement, stable and decreasing engagement, and one by a lower level of engagement: stable nonengagement, (3) adolescents who were younger, had no immigrant background, perceived more benefits of quitting smoking, and reported binge drinking preceding their baseline assessment were more likely to exhibit a stable engagement trajectory throughout the intervention, and (4) subjects who displayed a decreasing engagement pattern generally reduced their daily tobacco use more than subjects whose level of engagement was low.

This is the first study to examine engagement with a mobile phone–based smoking cessation intervention among adolescents. As expected, trajectories of higher and lower engagement were identified. We found similar results among adolescents as for adults [16,18,20]. We also identified a cluster of people who fully committed to the program, as in the study by Balmford and Borland [20]. Similarly, this study replicates three of the five engagement clusters detected by Christofferson et al [16]. Whereas our cluster-solution was less fine-grained, the clusters were significantly different with respect to baseline characteristics, contrary to those reported by Christoffersons et al [16]. Distinct groups are essential if interventions have to be adapted to different types of engager.

Furthermore, this study was the first to examine factors that predict stable engagement with a mobile phone–based smoking cessation program among adolescents. Other than expected from previous studies on mobile phone–based programs for adult smokers [15,16], engagement was not related to gender. This could be explained by the gender-specific tailoring which was undertaken for *MobileCoach Tobacco* (eg, the feedback on gender-specific drinking norms). Interestingly, in adolescents, being younger was associated with higher levels of engagement versus being older among adults [16]. This result suggests a quadratic relationship between age and engagement. Younger and older people might become more engaged for a variety of reasons that include the program being more novel to them, having more free time, or being more likely to commit to tasks in general. Contrary to our assumptions based on studies in adults, lower rates of daily cigarette consumption were not associated with higher engagement.

This study revealed three further factors, besides age, to be predictive of engagement among adolescents: the individual’s immigrant background, their personal outcome expectancies with respect to quitting smoking (ie, the benefits of quitting), and whether or not they previously engaged in binge drinking. An association between immigrant background and use of the program also was identified in a study by Businelle et al [11] that investigated the feasibility and effectiveness of an app-based smoking cessation intervention among socioeconomically disadvantaged adults. Especially non-white participants used the two information-delivering features of the app, which were tips and information about medication for quitting. Future studies should investigate whether tailoring mobile phone–based interventions to a person’s immigrant background impacts the intervention’s effectiveness. In particular, it has to be examined whether immigrants show less engagement with mobile phone–based programs because of poorer lexical-grammatical skills [34] or because of different interests and socialization than nonimmigrants.

Compared with previous research, the current findings underline the relevance of hazardous alcohol use in predicting engagement with a smoking cessation program. Recent studies on tobacco interventions [35-37] have already highlighted the underestimated role of combined alcohol and tobacco use among adolescents and its association with intervention effectiveness. Not only might mobile phone–based smoking cessation programs be more effective in adolescents who smoke and binge drink [9,35-37], they also could be more attractive to those adolescents. As such, measures are needed to make smoking cessation programs more attractive for adolescents who smoke but do not drink excessively.

Contrary to previous work on adult smokers [15,16,18], we were not able to certainly discern whether more engaged subjects were more likely to be cigarette abstinent after the intervention. This was because of the different reach at follow-up of more engaged compared with less engaged participants. The only stable finding over all statistical assumptions was that a decreasing engagement trajectory was associated with a greater reduction in daily tobacco use than a stable nonengagement trajectory. This result suggests that not only stable engagement but also decreasing engagement might be an indicator of behavior change. As illustrated by other studies [15,20], disengaging from an intervention might not necessarily mean disengaging from behavioral change. Instead, it could indicate a shift from extrinsic to intrinsic motivation [38]. These results support the claim by Yardley et al [39] to examine ways of improving “effective engagement” in subjects rather than simply more engagement, with “effective engagement” defined as sufficient engagement with the treatment to achieve intended outcomes.

One major challenge of future research, however, will be to sort out which kind of intervention is apt for nonengagers. One starting point will be to adapt smoking cessation programs for adolescents to address immigrant backgrounds and drinking behaviors to prevent stable nonengagement and thereby, potentially enhance treatment effectiveness. Considering that most stable nonengagers were more highly motivated to quit smoking at baseline than most stable engagers, one question to answer will be whether actions must be undertaken to increase active participation or not.

### Limitations

The findings of this study must be interpreted in view of its limitations. First, only answers to weekly prompts were included for engagement analysis. Other components of the program—such as downloading media content, setting a quit date, and extracurricular texting behaviors—were not included in our analyses; these components could all be analyzed to determine their own predictive values, similar to [15,40]. However, our rationale for selecting answers to prompts that were identical for all participants was to render our intergroup comparisons more interpretable. Second, that answers to weekly prompts were rewarded with 0.50 Swiss francs to cover the expenses of the adolescents might have influenced the adolescents’ likelihood of responding. Third, as already emphasized by Heminger et al [15], quantity and quality of answers to prompts could qualitatively differ (eg, a smoker who replies to all smoking-related prompts and indicates greater daily use of cigarettes). Rather than just analyzing registered events, future qualitative work should investigate whether the content of answers is associated with treatment outcomes. In addition, qualitative research should further investigate the different forms of motivation underlying engagement trajectories among smokers. As stated elsewhere [11], some highly engaged participants might have seen the program as integral to maintaining abstinence, whereas other nonabstinent smokers may have remained highly engaged to prepare for future smoking cessation attempts or merely to receive the offered remuneration. Finally, this study relied on self-report data of smoking behavior, which bears the risk that the results may have been influenced by social desirability.

### Conclusions

In summary, in our study, adolescents who smoked engaged to a large extent with a mobile phone–based smoking cessation program, irrespective of their initial intention to quit smoking. Decreasing engagement was in turn clearly associated with better long-term treatment outcomes. Further efforts should be undertaken to increase program engagement among older smokers, with an immigrant background, who do not drink excessively. In addition, future studies should not only examine the use of specific program components but also users’ engagement trajectories to better understand the mechanisms behind behavioral change.

## Abbreviations

ASW: average silhouette width
CCA: complete case analysis
CPD: cigarettes smoked per day
CPW: cigarettes smoked per week
DE: decreasing engagement
HAPA: health action process approach
ITT: intention-to-treat
LOCF: last-observation-carried-forward
OM: optimal matching
OR: odds ratio
RCT: randomized controlled trial
SD: standard deviation
SE: stable engagement
SE: standard error
SNE: stable nonengagement
